# Ethnobotanical survey of herbs used in the preservation of food products in Fez, Morocco

**DOI:** 10.1186/s42779-022-00144-5

**Published:** 2022-07-23

**Authors:** Yassine Ez zoubi, Sanae Lairini, Soukaina El Amrani, Fouad El-Akhal, Abdellah Farah, Rabia Bouslamti, Abdelhakim El Ouali Lalami

**Affiliations:** 1 Biotechnology, Environmental Technology and Valorization of Bio-Resources Team, Department of Biology, Faculty of Sciences and Techniques Al-Hoceima, Abdelmalek Essaadi University, Tetouan, Morocco; 2 Laboratory of Applied Organic Chemistry, Faculty of Sciences and Technologies, Sidi Mohamed Ben Abdellah University, route D’imouzzer, P.O. box 2202, Fez, Morocco; 3 Materials, Processes, Catalysis and Environment Laboratory, Higher School of Technology of Fez, Sidi Mohamed Ben Abdellah University, 30000 Imouzzer Road, Fez, Morocco; 4 Institute of Nursing Professions and Health Techniques of Tetouan (annex Al Hociema), Regional Health Directorate, Mohamed V Hospital, 32000 Al-Hoceima, Morocco; 5 Higher Institute of Nursing Professions and Health Techniques of Fez, Regional Health Directorate Fez-Meknes, El Ghassani Hospital, 30000 Fez, Morocco

**Keywords:** Ethnobotany survey, Herbs and spices, Food preservation, Herbalist, Fez city, Morocco

## Abstract

Morocco has a rich plant biodiversity and an ancient ethnobotanical knowledge influenced by the ethnic diversity of the Moroccan population. This heritage has been preserved from generation to generation by traditional herbalists. In this study, data were collected via a survey of herbalists based on the direct interview technique. Questions addressed the herbalists’ age and education level as well as the vernacular names and parts of the plants used for the preservation of food. The species use value was used to identify the most important species recommended by herbalists. The average age of the participants was 52.55 years, and the illiteracy rate was 46%. A total of 32 plant species representing 16 families were identified as being used in the preservation of various food products. The most important family was Lamiaceae, followed by Apiaceae and Rosaceae. The most frequently used parts of the plant were the leaves (26.37%), followed by the fruit (24.7%), and the whole plant (12.7%). The highest use values were recorded for *Citrus limon* (0.16), *Thymus vulgaris* (0.14), *Rosmarinus officinalis* (0.12), *Artemisia herba alba* (0.11) and *Lavandula dentata* (0.1). Meat, vegetables and fish were the most commonly preserved food products. This ethnobotanical survey on the preservation of food products is the first of its kind conducted in Morocco and has implications for meeting consumer demands and understanding the potential uses of plants in the preservation of food products.

## Introduction

Chemical preservatives can prevent food microbial deterioration. However, these synthetic products may be toxic and have harmful side effects. For example, there is increasing concern about the emergence of multidrug-resistant microbes due to the use of chemical preservatives. Consumers are increasingly interested in natural and healthy products [1]. Thus, many researchers are investigating natural substances, such as microbial metabolites, plants, and spice extracts, that have similar or better effects compared with synthetic additives [2, 3]. People have a long history of using traditional medicinal and aromatic plants for medical, food and cosmetic purposes. In all ancient civilizations and on all continents, we find traces of this use [4]. Thus, even today, despite advances in food preservation technologies, the traditional use of plants in food preservation has always occupied an important place in the food traditions of indigenous populations.

In this sense, the GIAHS project was adopted with the objective of contributing to the conservation of agricultural biodiversity, knowledge of systems and food, the assurance of livelihoods and culture. ''Traditional knowledge'' is a term that interchangeably means as indigenous knowledge is used to describe any information, knowledge, innovation or practice of local indigenous communities that is relevant to ensuring the conservation and sustainable use of biodiversity [5–7]. Throughout history, communities with strong ties to environmental dynamics have developed knowledge, practices, institutions and beliefs to adapt to recurring disruptions in securing their livelihoods [8]. This knowledge is also collected and documented by ethnobotanical studies to preserve the cultural heritage of the populations. Documentation of this information is helpful in recording local cultural traditions and enabling us to access important information [9].

Herbs and spices have long been used to flavour, colour and preserve foods and in culinary preparation in many parts of the world [10]. The flora in Morocco comprises more than 4200 species and subspecies belonging to 130 families and 940 genera. There are 800 endemic species (nearly 19% of the Moroccan flora), which helps to contextualize the unique ethnopharmacological and ethno-culinary heritage in Morocco. The ethnobotanical and ethnopharmacological study of Moroccan medicinal plants reveals their relative importance in the health system in Morocco. Several ethnobotanical studies have conducted on the use of Moroccan plant heritage in the pharmacological, industrial, cosmetic, spiritual, and food fields. The first serious study of Moroccan medicinal plants was a book entitled ‘Traditional medicine and West-Saharan toxicology: contribution to the study of the Moroccan pharmacopoeia’ by Jamal Bellakhdar, published in 1978 [11]. Since its release, several studies have focussed on plants traditionally used by the Moroccan population to treat to treat specific diseases or for cosmetic and spiritual uses. For example, a survey was conducted on the plants used to treat oral problems [12], diabetic diseases [13] and skin burns [14] in Rabat (in central Morocco), as a bio-insecticide against insect responsible for vector-borne diseases in the city of fez in central Morocco [15], to remedy COVID-19 at the level of the prefecture of Salé, north-western Morocco [16]. One bibliographic survey examined ethnobotanical studies conducted in Morocco between 1991 and 2019 to inventory the plants traditionally used against cancer [17]. Chaachouay et al. [18] investigated the different plants traditionally used in the Rif in northern of Morocco for the treatment of metabolic diseases such as diabetes, anaemia, hypercholesterolemia, obesity, and hyperthyroidism. Another study investigated the indigenous knowledge used to treat neurological diseases in the same region of Morocco (the Rif) [19]. In southern Morocco (Tarfaya province), 130 aromatic and medicinal plants spread over 57 were found to be used by the local population for the treatment of several diseases [20]. In an ethnopharmacological survey conducted in Taounate in northern Morocco, researchers identified 102 plants used to treat several illnesses [21]. In the north-eastern region of Morocco, 55 species of plants belonging to 36 families were identified as potentially toxic plants in an ethnobotanical study by Kharchoufa et al. [22]. Despite the existence of vibrant culture and ancient traditions to preserve crops and seasonal foods, no ethnobotanical study has been conducted to investigate the plants and spices traditionally used by the Moroccan population to preserve foods.

Fez (in Arabic: فاس, Fās; in Berber:  , Fas) is a city in northern Morocco, 180 km east of Rabat (the capital of Morocco). Fez is located in the Saïs plain, between the Rif to the north and the Middle Atlas to the south. As part of the imperial cities of Morocco, Fez was the capital of the country during several periods and is considered today as its spiritual capital. It extends over three sectors: the royal enclosure, the new districts, and the old town (Medina), which is a UNESCO World Heritage Site and evokes mediaeval towns with its streets, gates, walls, and shops grouped by specialty [23]. Fassi cuisine is diverse and is the result of centuries of exchange and openness to other peoples, with particularly marked Slavic, Persian, and Andalusian influences. The city is well known for its rich craftsmanship, where knowledge is jealously guarded and transmitted from father to son for generations. Indeed, the population of Fez has a rich and ancient tradition in the field of phytotherapy. It is an Andalusian and Arab-Berber heritage, largely influenced by the Islamic religion, and uses medicinal plants in the treatment of several diseases, to flavour meals, and for spiritual purposes. The herbalists in the city of Fez are distributed in specialized districts, where they give herbal remedies and advice as they sell the dried plants whole and crushed, and sometimes fresh.

The Quarawiyine University in Fez, one of the oldest universities in the world, was the academic centre of Africa and included a medicine section. This university has made it possible to safeguard the traditional culture of medication and the use of traditional medicines. Based on ethnobotanical research on the use of aromatic and medicinal plants in Fez [24, 25], we can assume that the Fez region has preserved a vibrant heritage around the use of plants and spices for traditional food preservation.

To our knowledge, a detailed ethnobotanical study of plants used for food preservation in Morocco has not been reported so far. Considering the traditional heritage and the culinary experience known among herbalists in the city of Fez, we tried to investigate the use of medicinal plants by herbalists in the city of Fez, providing data on the indications of the species used and their applications as biopreservatives. Indeed, it is very important to transform this traditional knowledge from the herbalists into scientific knowledge through research on bioactive molecules in the preservation of food in order to evaluate, preserve and transmit this traditional heritage. The Fez area was selected as a first step for implementing a more systematic survey for plants preservation of foods in Morocco in the future.

## Material and methods

### The area of the study

Fez is located in the northern half of Morocco between 5°07′00ʺ and 4°55′00ʺ W and 34°00′00ʺ and 34°05′00ʺ N. The city is at the eastern end of the Saïs basin which itself is part of the Southern Rif Furrow. The urban perimeter of the city covers an area of approximately 450 km^2^. The climate is Mediterranean, with mild and humid winters and extremely dry conditions from June to September.

### Ethnobotanical survey

In this study, data were collected via a survey of herbalists based on the direct interview technique. Questions focussed on the different plants recommended by the herbalists for clients to preserve food products. The study took place between March and July 2017; during this period, 300 interviews were conducted with herbalists of both genders out of 700 herbalists practicing legally and registered with local authorities. A total of ten questions with the herbalists through these questionnaires, information was collected regarding the participant population into direct questions (age, gender, and level of education). Two closed questions (yes/no) on the authorization of the exercise of this activity by the authorities, and if he has knowledge of plants used as food preservatives. Open questions with the herbalists were collected including local name, preserved products, plant parts used, and duration of the preservation. The data collection time varies between 30 and 40 min for each herbalist.

**Fig. 1:**
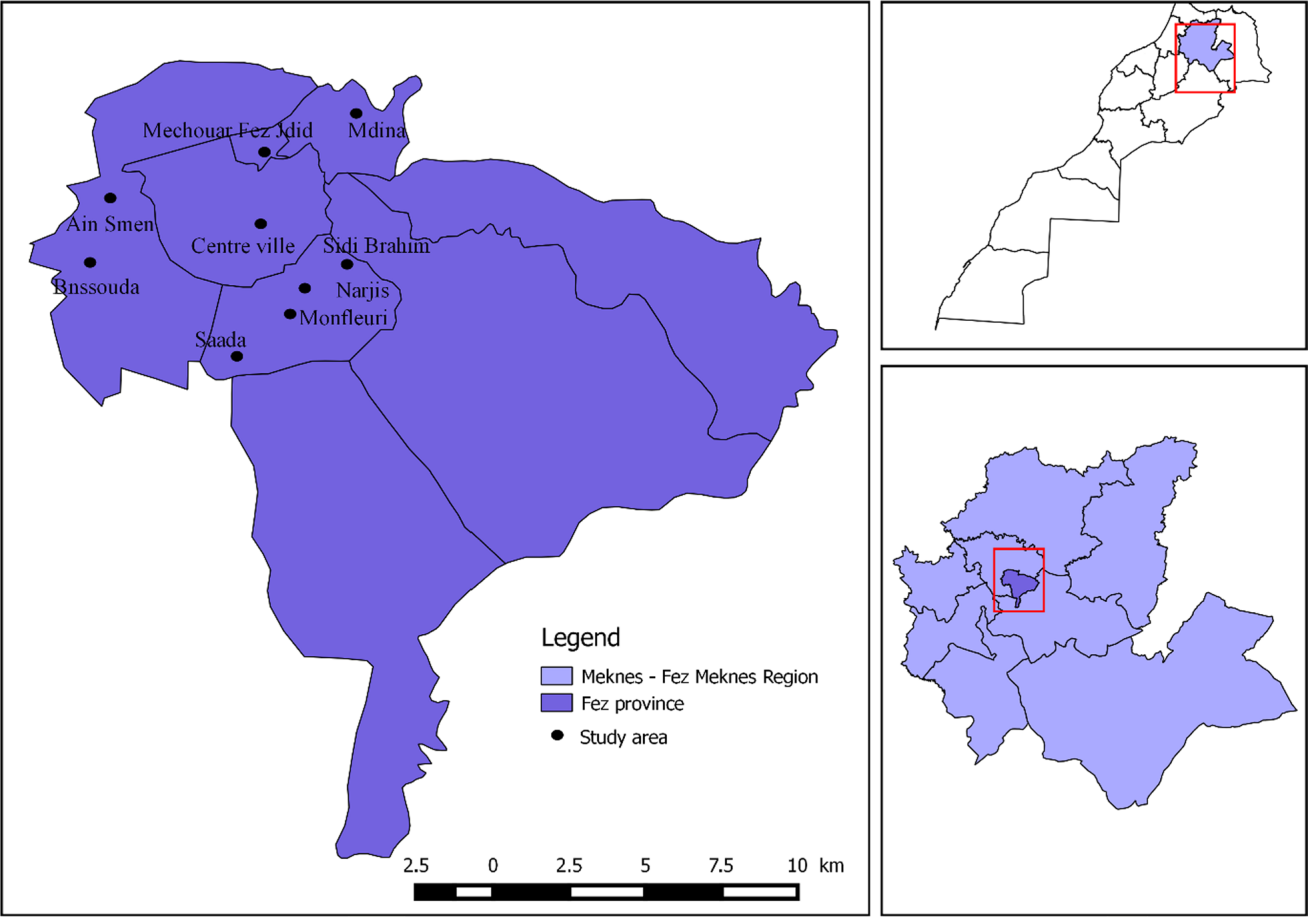
Map of the study area in the Fez city, Morocco

Data were divided into 9 groups that correspond to the number of the urban boroughs of Fez; all districts studied belong to the urban area of Fez (Table 1 and Fig. 1).

**Table1:** Distribution of investigations by each group

Groups	Boroughs	Number of surveys
Group 1	Ain Smen	32
Group 2	Bnssouda	28
Group 3	Centre ville	29
Group 4	Narjis	43
Group 5	Saada	40
Group 6	Sidi Brahim	27
Group 7	Mdina	38
Group 8	Monfleuri	30
Group 9	Mechouar Fez Jdid	33
Sample (total)		300

During this study, ethical provisions were taken into consideration, namely:

- We included herbalists practicing in a legal manner in the city of Fez who agreed to join the study;
- Membership in the survey was free and unconstrained, and any participant could voluntarily stop participating at any time during the study;
- Herbalists who did not have an official place to sell their products were not included in the study;
- We respected anonymity and confidentiality relating to the identity of the participants;
- The participants were informed about the purpose and framework of the study as well as the subsequent use of the results.

### Identification of plant species

The samples of plants and spices listed were collected during several visits to the herbalists interviewed in the different regions of the study area. Taxonomic identification was performed in the laboratory according to the manuals for the determination of vascular plants ‘Practical flora of Morocco’, Volumes 1, 2 and 3 [26–28]. Verification and confirmation of the species were conducted by Professor Abdelkader Taleb in the department of Natural Resources & Environment, Hassan II Institute of Agronomy and Veterinary Medicine, Rabat. All specimens were deposited in the herbarium of the National Institute of Aromatic and Medicinal Plants in Taounate.

### Species use value (SUV) determination

The species use value, a quantitative method that demonstrates the relative importance of plant species known locally, was also evaluated according to the following formula [29]:

$${\text{UVi}} = \sum {{\text{Ui}}/{\text{Ni}}}$$

where UVi refers to the use value of a species, Ui to the number of citations per specific plant species, and Ni to the number of herbalists. A high species use value indicates the potential importance of the plant species reported.

### Statistical analysis

The information obtained during the interviews was statistically analysed using Microsoft Office Excel software (2010).

## Results

### Uses of plants according to gender and education level

A total of 300 herbalists (206 men [68.66%] and 94 women [31.33%]) between 26 and 86 years of age were interviewed. The average age of the participants was 52.5 years. The illiteracy rate was 46%; 18% had a primary-level education, 17% secondary level and 19% university level (Table 2).

**Table 2:** Demographics information of the herbalists in the Fez city

Variable	Category	Number of informants (*N* = 300)	%
Sex	Female	94	31.33
	Male	206	68.66
Age group (year)	Between 18 and 20	0	0
	21–30	22	7.33
	31–40	51	17
	41–50	73	24.33
	51–60	48	16
	61–70	49	16.33
	71–80	29	9.66
	> 80	28	9.33
Educational level	Illiterate	138	46
	Primary	54	18
	Secondary	51	17
	University	57	19

### Medicinal plant diversity and plant parts used

**Fig. 2:**
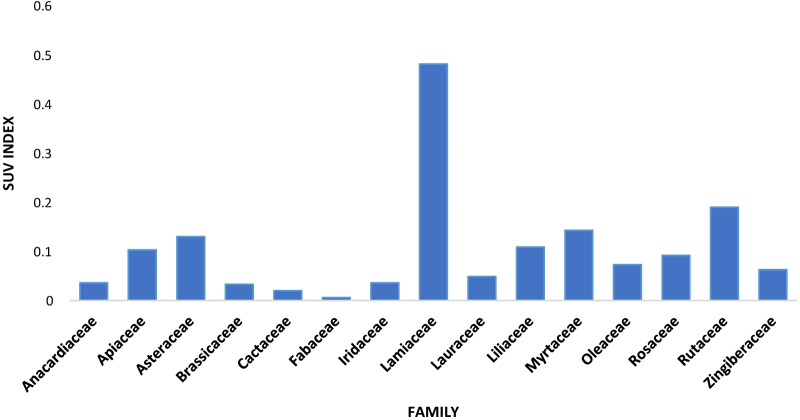
Graph of the main effects of the average SUV index according to the family

The present study found 32 plant species belonging to 16 plant families that were used in the preservation of different types of food products in Fez. Table 3 lists the utilised medicinal plant species arranged in alphabetical order by family name, scientific name, local name, number of citations, preserved food product, duration of the preservation, and species use value. The results reveal that the taxonomic family with the greatest number of utilised plants was Lamiaceae (eight species), followed by Apiaceae and Rosaceae (three species each) (Table 4). The remaining plant families were represented by only one or two species. This indicates the widespread importance of the abovementioned families in the study area. Species use values (SUV) indicate the relative importance of plant species among practitioners (Table 3 and Fig. 2). The highest SUV values were recorded for *Citrus limon* (0.16), *Thymus vulgaris* (0.14), *Rosmarinus officinalis* (0.12), *Artemisia herba alba* (0.11) and *Lavandula dentata* (0.1).

**Table 3:** Enumeration of medicinal plants used in preservation of foods in Fez city

Family	Voucher number	Botanical name	Local name	Preserved product	The duration of the conversation	Number of citations	SUV
Lamiaceae	ANL0010	*Lavandula angustifolia* Mill	Khzama_خزامى	Butter	Three months	32	0.1
	ANL0003	*Mentha pulegium* L	Fliou_فليو	Milk	One week	20	0.06
	ANL0015	*Origanum vulgare* L	Saatar_سعتر	Fig	Several years	10	0.03
	ANL0023	*Rosmarinus officinalis* L	Azir_أزير	Meat	Several years	37	0.12
	ANL0052	*Salvia officinalis* L	Salmia_سالمية	Meat	One year	5	0.016
	ANL0120	*Thymus vulgaris* L	Ziitra_زعيترة	butter	Three months	43	0.14
	ANL0122	*Marrubium vulgare* L	Mriwa_مريوة	Meat	One year	3	0.01
	ANL0004	*Mentha spicata* L	Minta_مانتة	Vegetables	One month	2	0.006
Rutaceae	ANR0110	*Citrus limon* L	Limoun_ليمون	Olive	One year	48	0.16
	ANR0098	*Citrus aurantium* L	lranj_الرنج	Drinks	Three months	9	0.03
Apiaceae	ANA0063	*Cuminum cyminum* L	Kamoun_كمون	Meat and fish	One week	4	0.013
	ANA0045	*Foeniculum vulgare* Milll	Basbas_بسباس	Meat	One week	3	0.01
	ANA0061	*Coriandrum sativum* L	Kazbor_قزبر	Meat and butter	Three months	24	0.08
Rosaceae	ANR0125	*Rosa centifolia* L	Ward al baldi_الورد البلدي	Milk	One week	3	0.01
	ANR0220	*Prunus dulcis* Mill	Louz_اللوز	Drinks	One week	8	0.026
	ANR0093	*Malus domestica* Borkh	Tfah_التفاح	Vegetables	Several years	17	0.056
Liliaceae	ANLI025	*Allium sativum* L	Toum_ثوم	Meat	One week	29	0.096
	ANLI024	*Allium cepa* L	Basla_بصل	Meat	One week	4	0.013
Asteraceae	ANAS006	*Artemisia herba alba* Asso	Chih_شيح	Meat	One year	34	0.11
	ANAS014	*Cynara cardunculus* L	Lquoqe_القوق	Milk	One week	6	0.02
Lauraceae	ANLA087	*Cinnamomum verum*	Quarfa_قرفة	Fruits	Three months	7	0.023
	ANLA93	*Laurus nobilis* L	Wraq sidna Moussa_أوراق سيدنا موسى	vegetables	One month	8	0.026
Myrtaceae	ANM0046	*Eucalyptus globulus* Labill	Kalitous_كاليتوس	Vegetables	One year	30	0.1
	ANM0055	*Syzygium aromaticum* L	Krenfel_القرنفل	Fruits	One month	13	0.043
Zingiberaceae	ANZ0063	*Curcuma longa* L	Kharquoum_ الخرقوم	Fish	One week	7	0.023
	ANZ0086	*Zingiber officinale* Roscoe	Skinjbir_ سكينجبير	Meat	One week	12	0.04
Anacardiaceae	ANA0102	*Schinus molle* L	Libzar_لبزار	Fish	One month	11	0.036
Brassicaceae	ANB0008	*Lepidium sativum* L	Hab rachad_حب الرشاد	Fish	One month	10	0.033
Cactaceae	ANC0074	*Opuntia ficus-indica* (L.) Mill	Handia_هندية	Drinks	One week	6	0.02
Fabaceae	ANF0120	*Trigonella foenum-graecum* L	Halba_حلبة	Meat	One week	2	0.006
Iridaceae	ANI0052	*Crocus sativus* L	Zaafran_زعفران	Meat and fruits	One week	11	0.036
Oleaceae	ANO0047	*Olea europaea* L	Zitoun_زيتون	Vegetables	Several years	22	0.073

**Table 4:** Taxonomic diversity of medicinal plants used for preservation food products in Fez city

Family	Number of species by family	Percentage of family (%)
Lamiaceae	8	25
Apiaceae	3	9
Rosaceae	3	9
Liliacae	2	6
Asteraceae	2	6
Lauraceae	2	6
Myrtaceae	2	6
Zingiberaceae	2	6
Anacardiaceae	1	3
Brassicaceae	1	3
Cactaceae	1	3
Fabaceae	1	3
Iridaceae	1	3
Oleaceae	1	3
Rutaceae	2	6

**Fig. 3:**
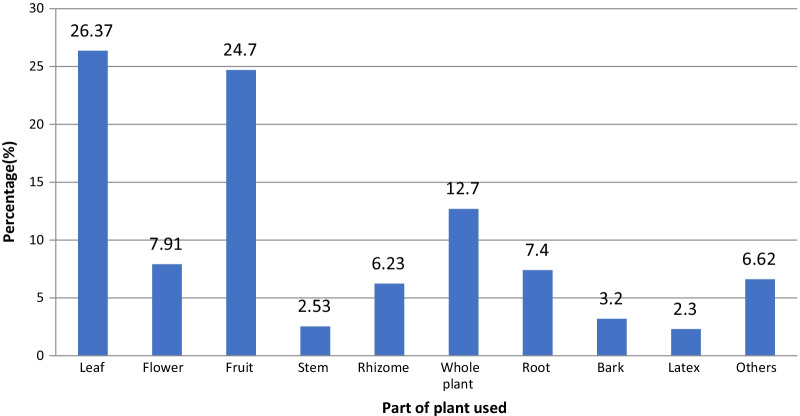
Different plant parts used by herbalists in Fez city

The most frequently used plant parts for preparations were leaves (26.37%), fruit (24.7%), whole plant (12.7%), flower (7.91%), root (7.4%), rhizomes (6.23%), bark, (3.2%), stem (2.53%) and latex (2.3%) (Fig. 3).

### Preserved food product and duration of the preservation

**Fig. 4:**
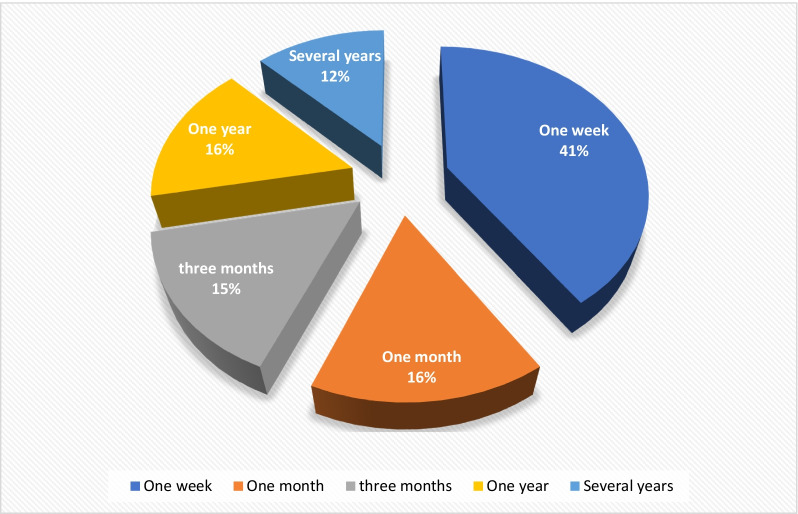
Percentage of duration of the food products conservation

As shown in Table 5, meat was the most preserved food product (34.28%), followed by vegetables (14.28%) and fish (11.42%), respectively. Butter, milk, drinks, and fruits were also preserved (8.57% for each). Most products were preserved for one week (41%), 16% for one month, and 16% for one year (Fig. 4).

**Table 5:** Frequency of preserved foods

Types of foods	Number of species used for preservation	Percentage (%)
Meat	12	34.28
Vegetables	5	14.28
Fish	4	11.42
Butter	3	8.57
Milk	3	8.57
Drinks	3	8.57
Fruits	3	8.57
Fig	1	2.85
Olive	1	2.85

## Discussion

Morocco has high cultural diversity, a rich traditional medical system and associated traditional knowledge, and high rates of biodiversity which combine to provide a diversity of medicinal plants. Traditional medical practice continues to serve a large clientele, and its prestige in the eyes of the mass remains virtually undiminished. The Union of Moroccan Herbalists (UMH) is a legislative framework that brings together herbalists practicing the profession in Morocco. However, no official statistics on their total number are available [30]. The profession of herbalist is governed by three law chapters, all of which date back to before 1960. The Dahir of 19 February 1960 establishes the criteria for herbalists to possess and sell all medicinal plants and parts, fresh or dried, with the exception of plants categorized in the numerous toxic substance’s tables established by the Dahir of 2 December 1922 [30]. Herbalists are a valuable source of information on medicines from plants. They have gained experience over a long period of practice, incorporating knowledge as a legacy of their ancestors from the Amazigh, Arab and sub-Saharan African cultures [31]. Knowledge of plants and their use to treat a particular condition is timeless. The work of the herbalist is based on experience and learning from a master or a family member doing the same job. The masters are therapists who have undergone training in basic Arbo-Muslim therapy books on plants therapy in ‘’Madrasa’’ or traditional universities such as those in Marrakech, Fes, and Tetouan, or medicine on ancient scholarly works (Avicenna, Ibn Zohr, Ibn Tofail, Ibn Buklarich, Ibn Al-Baytar, Kuhin Al-Attar), on oral heritage as well as on their own experience, which allows you to know the doses and the mode of use of the plants [32–34]. Some herbalists update and improve their knowledge in this way over time, others by reading books in the field. The herbalist is not only a seller of medicinal plants but also has the professional capacity to recommend the use of plants to his clients. The herbalist can also make preparations with several plants according to the requirements of the client.

In this study, the average age of the herbalists who participated was 52.55 years. This could be explained by the time necessary to acquire experience from their parents or other practitioners. Participating herbalists reported all levels of educational background, from the illiterate (46%) to the university-level scholar (19%). This result is compatible with the last general census of the population in Morocco (2014 census) which showed that the rate of illiteracy was 45%; 21.2% of the population had a primary-level education, 12.3% college-level, 10.2% high school-level, and 8% higher education [35].

In our study, the Lamiaceae family and species of *Citrus limon*, *Thymus vulgaris*, *Rosmarinus officinalis*, *Artemisia herba alba*, and *Lavandula dentata* were the most commonly used plants for traditional food preservation in Fez. The food preservation properties of many herb species have been studied to date. Studies investigating rosemary, cinnamon, bay, sage, garlic, oregano, and ginger have shown positive results for their capacity to act as preservatives of meat [36, 37]. Gutierrez et al. [38] demonstrated that a combination of *Origanum majorana* and *Thymus vulgaris* EOs had an additive effect against meat spoilage organisms than when either EO was used individually. Lavender EO has been tested for the preservation of produce, meat and fish. In the case of ground beef, lavender was found to be effective in preventing growth of *Escherichia coli*, a major contaminant in meat processing [39]. A study on the preservation potential of essential oil of *Citrus limon* (0.06 and 0.312 mg/g) on ground meat showed strong activity against *Listeria monocytogenes* during storage of meat at 4 °C [40]. The preservative power of rosemary extracts and essential oils has been amply demonstrated in several food models: beef meatballs, stewed beef, and pork sausage [41]. A study conducted by Gomez-Estaca et al. proved that the rosemary essential oil allowed the extension of the shelf life of the fish, and this by inhibiting the growth of common food bacteria in particular enterobacteria contributing to food spoilage [42]. Olmedo et al. [43] evaluated the effect of rosemary EO on the oxidative and fermentative stabilities of flavoured cheese prepared with cream cheese base. The authors found that rosemary EO demonstrated a protective effect against lipid oxidation and fermentation in cheese. In addition, Govaris et al. reported an inhibitory effect of dietary supplementation of turkeys with rosemary on the growth of bacteria responsible for meat spoilage. Camo et al. [44] also observed an inhibitory effect of the use of rosemary extract added to lamb meat packaged in a modified atmosphere on the growth of psychrotrophic bacteria. A study carried out by Cleonice Gonçalves et al. (2020) on the antioxidant and antimicrobial powers of *Origanum vulgare* and *Thymus vulgaris*, the nanocapsules based on the essential oils of these two plants have been proven to show physical and chemical stability for a period of 90 days’ storage. These nanocapsules have greater antimicrobial activity against gram-positive bacteria than gram-negative bacteria. The nanocapsules produced also exhibited high thermal resistance to baking processes, protecting the bread from the proliferation of moulds and yeast [45]. On the other hand, other plants suggested by herbalists in our study for their preservative power have not yet been studied in the preservation of food models, such as *Artemisia herba alba* Asso, *Syzygium aromaticum* and *Crocus sativus* L.

Medicinal plants are rich in terpenes and phenolic compounds that present antimicrobial and antioxidant properties; in addition, the literature shows that these bioactive compounds extracted from other plants have been effective in food systems [46]. Phenolic compounds (tannins, coumarins, and flavonoids) are known to alter microbial cellular permeability, resulting in loss of macromolecules, and interacting with membrane proteins, causing structural changes [47]. A simple phenol example is caffeic acid, which is found in thyme and tarragon and is active against fungi, viruses, and bacteria. Eugenol is a phenolic compound found in clove oil that is active against bacteria and fungi [48]. Some alkaloids from plants have also been used as antimicrobials in food [49]. Recently, many studies have investigated the antibacterial effect of phytochemical extracts against foodborne pathogens, which makes it possible to make healthy and efficient preservatives [50–52].

The results of this study confirm that the plant parts most commonly recommended by herbalists for preserving food were the leaves and fruits. In agreement with our results, several studies reported that the aerial parts of plants (leaves, flowers, and fruits) are most commonly used by the population to extract bioactive molecules [53–55]. The preferential use of the aerial part of plants could be due to the easy harvesting of this part of the plant compared to the roots. In addition, the leaves are the seat of most types of volatile molecules (monoterpenes, sesquiterpenes, aldehydes, ketones, and alcohols) and non-volatile phytochemicals (tannins, mucilages, flavonoids, etc.). The abundant use of the leaves, fruits and flowers of aromatic and medicinal plants by the local population also promotes the preservation of natural resources. Some wild medicinal flora is threatened by major factors such as urbanisation, unforeseen climatic changes, expansion of new agricultural lands, overgrazing and unscientific harvesting methods, which necessitates taking adequate measures to preserve the plant heritage through the use of domesticated plants such as Mentha, Citrus, Origan, and Lavandula.

The high preservation rate of meats (34.28%) could be explained by an annual religious holiday called Eid al-Adha, one of two main Islamic holidays, which is characterised by the sacrifice of a sheep or a goat of at least one year of age. The Moroccan population consumes several traditional meat products (Kaddid and Khlii), which are transformed according to traditional processes, inducing biochemical and microbial changes which increase the shelf life while improving the flavour and the nutritional quality of these products. Several studies have proven the protective effect of essential oils and hydroalcoholic extracts to fight against pathogenic bacteria contaminating various meat matrices. Khaleque et al. evaluated the effect of cloves (*Syzygium aromaticum*) and cinnamon (*Cinnamomum cassia*) on *Listeria monocytogenes* in ground beef stored at different refrigeration temperatures. The results demonstrated that 10% of *S. aromaticum* and *C. cassia* essential oils could completely inactivate *L. monocytogenes* in ground beef within 3 days of inoculation, regardless of storage temperature [56]. Thirty-five phenolic compounds (three stilbenes, eight cinnamic acids, six benzoic acids, 11 flavonoids, five coumarin and two naphthoquinones) at a concentration of 1 g/L were examined against six pathogenic bacterial strains of food origin or harmful to food: three Gram-positive (*S. aureus, B. subtilis and L. monocytogenes*) and three Gram-negative (*E. coli, P. aeruginosa and S. Enteritidis*) [57]. The bacterial load difference (BLD) was used to determine the effect of bacterial growth of each of the polyphenols studied. The authors found that *L. monocytogenes* was sensitive to all tested polyphenols, with 54.3% compounds having a BLD above 50%. Stilbenes, resveratrol, and pinosylvin were very active against *L. monocytogenes,* with BLDs of 100% and 97.9%, respectively. Cinnamic acids (both cinnamul-3,4-dihydroxy-*α*-cyanocinnamate and CU-CPT22) had BLDs of 100%, followed by caffeic acid 1,1-dimethylallyl ester (BLD of 72.3%) and chicoric acid (BLD of 53.5%). Butyl gallate was also highly active with a BLD of 100%, followed by 3,4-dihydroxy-benzoic acid methyl ester with a BLD of 58.6%. The BLD values of flavonoids compounds (epigallocatechin gallate, quercetin 3-*β*-D-glucoside, taxifolin, myricitrin, and cardamomin compounds) were 100%, 75.1%, 69.4%, 58.5%, and 51.7%, respectively. All studied coumarins, except 5,7-dihy-droxy-4-propylcoumarin, showed activity against the *L. monocytogenes*, and naphthoquinones had a BLD of 100%. In another study, the luteolin (flavones) isolated from *Rumex tingitanus* were been found to be responsible for antibacterial activity against *L. monocytogenes* in minced beef meat [58].

The application of EOs and phenolic extracts as bioconservative agents in food products must be considered in the context of their organoleptic suitability and potential toxicological effects. Although plant extracts are natural compounds that have been used since ancient times, their use does not imply that they are harmless. It is necessary to assess important factors such as their dose, efficacy, and toxicity. Therefore, before incorporation into meat products, in vitro and in vivo toxicity studies as well as bio-accessibility and bioavailability tests will be essential to understand their behaviour in the human body [59].

## Conclusion

The present study found 32 herbs and spices belonging to 16 families that are used in the preservation of different types of food products in Fez, Morocco. The results revealed that the taxonomic family with the greatest number of utilised plants was Lamiaceae (eight species), followed by Apiaceae and Rosaceae (three species each). The highest SUV values were recorded for *Citrus limon*, *Thymus vulgaris*, *Rosmarinus officinalis*, *Artemisia herba alba*, and *Lavandula dentata*.

The plants listed could be a source of molecules for a new generation of bioconservatives. In this context, our work is currently focussing on evaluating the antioxidant and antibacterial benefits of several bioactive molecules extracted from these plants, in particular, the antioxidant effects of *Crocus sativus* L. (saffron) extracts enriched with crocin, picrocrocin, and safranal. Other work to evaluate the bioconservative role of phenolic extracts and essential oils of the plants identified in this study could also be promising.

This work is a contribution to the documentation of traditional local knowledge in the field of food preservation. The results of this study give new credibility and status to the informal knowledge of local herbalists. In this sense, more in-depth studies on the sources of information of herbalists, on their ethnic categories, and the modes of transmission of their knowledge from one generation to another could be very interesting to shed light on the process of transmission of knowledge between the generations.

## Data Availability

All data generated or analyzed during this study are included in this published article.
